# Pharmacological Applications of Nrf2 Inhibitors as Potential Antineoplastic Drugs

**DOI:** 10.3390/ijms20082025

**Published:** 2019-04-24

**Authors:** Pelin Telkoparan-Akillilar, Sibel Suzen, Luciano Saso

**Affiliations:** 1 Department of Medical Biology, Faculty of Medicine, Yuksek Ihtisas University, 06520 Balgat, Ankara, Turkey; pelinta@yiu.edu.tr; 2 Department of Pharmaceutical Chemistry, Faculty of Pharmacy, Ankara University, 06100 Tandogan, Ankara, Turkey; sibel.suzen@pharmacy.ankara.edu.tr; 3 Department of Physiology and Pharmacology, “Vittorio Erspamer”, Sapienza University of Rome, P. le Aldo Moro 5, 00185 Rome, Italy

**Keywords:** Nrf2 inhibitors, antineoplastic drugs, cancer, chemoresistance, cancer chemoprevention and therapy

## Abstract

Oxidative stress (OS) is associated with many diseases ranging from cancer to neurodegenerative disorders. Nuclear factor-erythroid 2 p45-related factor 2 (Nrf2) is one of the most effective cytoprotective controller against OS. Modulation of Nrf2 pathway constitutes a remarkable strategy in the antineoplastic treatments. A big number of Nrf2-antioxidant response element activators have been screened for use as chemo-preventive drugs in OS associated diseases like cancer even though activation of Nrf2 happens in a variety of cancers. Research proved that hyperactivation of the Nrf2 pathway produces a situation that helps the survival of normal as well as malignant cells, protecting them against OS, anticancer drugs, and radiotherapy. In this review, the modulation of the Nrf2 pathway, anticancer activity and challenges associated with the development of an Nrf2-based anti-cancer treatment approaches are discussed.

## 1. Introduction

Cancer is the second leading cause of death both for men and women, behind cardiovascular diseases [1]. According to the World Health Organization (WHO), 9.5 million people died of cancer, mostly in low- and middle-income countries, in 2018 [1]. New cancer cases are expected to rise about 64% worldwide by 2040 [1]. During carcinogenesis, a normal cell evolves into a tumor cell, which is a multi-stage process involving multiple epigenetic and genetic events in three stages: initiation, promotion, and progression [2]. Cancer is still a major threat to our health, despite the extensive research efforts to develop new treatments. Hence, it is necessary to develop novel strategies to improve the outcomes of patients suffering from aggressive or treatment-resistant malignancies. Recent studies have showed that oxidative stress (OS) is one of the crucial causes responsible for cancer and may lead to tumor aggressiveness, malignant progression and resistance to treatment [3].

There are many types of cancer treatment. The types of treatment that that patient will receive will depend on the type of cancer and how advanced it is. Today, we can talk about surgery, radiotherapy, chemotherapy, immunotherapy, targeted therapy, hormone therapy and stem cell transplants processes that are there to treat cancer. In addition, precision medicine helps doctors select treatments that are most likely to help patients, based on a genetic understanding of their disease.

Types of immunotherapy that help the immune system act directly against the cancer include: Checkpoint inhibitors, adoptive cell transfer, monoclonal antibodies, treatment vaccines, cytokines, BCG (Bacillus Calmette-Guérin). Although there are good advantages, immunotherapy is not yet as widely used as surgery, chemotherapy, and radiation therapy. Many new immunotherapies are being studied in clinical trials [4,5].

Targeted therapy is the foundation of precision medicine. Most targeted therapies are either small-molecule drugs or monoclonal antibodies. Generally, targeted therapies help the immune system destroy cancer cells, stop cancer cells from growing, stop signals that help form blood vessels, deliver cell-killing substances to cancer cells, cause cancer cell death, starve cancer of the hormones it needs to grow. The important drawbacks of targeted therapy include resistance of cancer cells to the therapy and difficulties of developing drugs to some targets [6,7].

Stem cell transplants are most often used to help people with leukemia and lymphoma. They may also be used for neuroblastoma and multiple myeloma. Stem cell transplants for other types of cancer are being studied in clinical trials [8,9].

Precision medicine may be called personalized medicine. The idea of this treatment is to develop a treatment that will be tailored to the genetic changes in each person’s cancer. However, the precision medicine approach to cancer treatment is not yet part of routine care for most patients [10,11].

OS plays a crucial role in determining cell fate. As a reaction to the excessive reactive oxygen species (ROS) load, apoptotic-signaling pathway is stimulated to promote normal cell death. Nuclear factor-erythroid 2 p45-related factor 2 (Nrf2) looks as if to be as a chief regulator, which defends cells [12]. Nrf2 is usually degraded in cytoplasm by interaction with Keap1 inhibitor. However, excess amount of ROS stimulates tyrosine kinases to separate Nrf2. Deregulation of Nrf2 and/or Keap1 due to mutation and stimulated upstream oncogenes is related with nuclear accumulation and activation of Nrf2 to protect cells from apoptosis and induce proliferation, metastasis and chemoresistance. Nrf2 modulation appears to be significant in the personalization of cancer therapy [13]. In this review, we focus our attention on the role of Nrf2 in cancer progression and pharmacological applications of Nrf2 inhibitors as potential antineoplastic drugs.

## 2. Nrf2 Domains and Their Functions

Nrf2 (also known as NFE2L2) belongs to the cap ’n’ collar type of basic region leucine zipper factor family (CNC-bZip) that is a group of transcription factors that are activated in response to cellular stress [14]. Nrf2 is the most-known CNC family member and regulates the expression of antioxidants phase I-II metabolizing enzymes and endogenous antioxidants [15]. The human Nrf2 gene was first identified and characterized in 1994, which encodes a protein of 605 amino acids [14,16]. Nrf2 has highly conserved seven functional domains, called Nrf2-ECH homology (Neh1 to Neh7) [12]. Neh1, Neh3 and Neh6 domain are located in the C-terminal region. Neh1 comprises a conserved CNC-bZIP region binds to antioxidant responsive elements (AREs), which are crucial for the transcriptional activity of Nrf2, and it is also needed for homo-hetero dimerization with Maf proteins (MafF, MafG and MafK) [12]. The Neh2 domain is located at the N-terminal of the Nrf2 and it contains DLG and ETGE motifs. Kelch-like ECH-associated protein 1 (Keap1) binds directly to these motifs and negatively regulates Nrf2 levels via proteasomal degradation of excess protein under homeostatic conditions [16,17]. Neh3, 4, and 5 are known transactivation domains that are involved in transactivation by transcription factors. The Neh3 domain is present at the C-terminal regions and binds to chromo-ATPase/helicase DNA binding protein 6 (CHD6) [18]. The Neh4 and Neh5 are transactivation domains that bind cAMP response element binding protein (CREB) and/or the receptor-associated co-activator (RAC) [19]. The Neh6 domain is localized between Neh7 and Neh1 domains, and it plays a key role in the Keap1-independent degradation of Nrf2 by recruiting the dimeric β-transducin repeat-containing protein (β-TrCP) under redox stress conditions [20]. The most recently identified domain of Nrf2 is Neh7, which interacts with the retinoid X receptor alpha (RXRα) and inhibits the NRF2–ARE signaling pathway [21].

## 3. Regulation of Nrf2 Signaling Pathway

**Figure 1:**
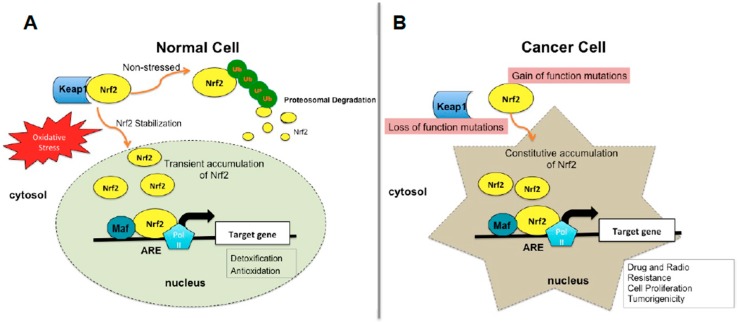
Nrf2 status in normal and cancer cells. (**A**) In a normal cell, under normal conditions Keap1 inhibits transcriptional activity of Nrf2 via ubiquitination and proteasomal degradation, under stress conditions Keap1 and Nrf2 interaction does not occur, causing Nrf2 stabilization and accumulation in nucleus, which in turn induces cytoprotective gene expression. (**B**) In a cancer cell, somatic mutations in NRF2 (gain of function mutations) and KEAP1 (loss of function mutations) result in constitutive activation of Nrf2 inducing expression of genes related to tumorigenity. Nrf2, nuclear factor erythroid 2-related factor 2; Keap1, Kelch-like ECH-associated protein 1; Maf, small musculoaponeurotic fibrosarcoma protein; Pol II; Polimerase II; ARE: Antioxidant response element

The Nrf2 signaling pathway contributes to the maintenance of cellular and tissue homeostasis and protects cells against OS. The repressor protein Keap1, an adaptor component of Cullin 3-based ubiquitin E3 ligase complex, tightly regulates the activities and the protein level of Nrf2 [22]. Under basal homeostatic conditions, Nrf2 is localized in the cytoplasm and binds to its inhibitor protein Keap1, which inhibits transcriptional activity of Nrf2 via ubiquitination and proteasomal degradation [23]. Under stress conditions Keap1 cysteine residues are modified with thiols and proteasomal degradation of Nrf2 is inhibited. As a consequence, Nrf2 dissociates from Keap1 and translocates into the nucleus, forms heretodimers with small Maf proteins and induces transcription of cytoprotective gene expression, such as NADPH quinone oxidoreductase (NQO-1), glutathione S-transferases (GSTs), heme oxygenase-1 (HMOX1), and glutamate-cysteine ligase (GCL) subunits after ARE-sequence binding (Figure 1A) [23,24,25]. Although, Keap1 is the main regulator of Nrf2, there are other alternative pathways that can impact Nrf2 activity. For example, glycogen synthase kinase 3 (GSK-3β) phosphorylates serine amino acid residues located in the Neh6 domain that directs to Nrf2 ubiquitination and proteosomal degradation in Keap1 independent manner [26]. In addition, researchers showed that a novel E3 ubiquitin ligase, Hrd1, interacts with the Neh4/Neh5 domains of Nrf2, which results in enhanced Nrf2 ubiquitylation and degradation [27]. Studies demonstrate that Nrf2 is also regulated at the transcriptional level. Oncogenic KRAS, BRAF and C-MYC can increase the mRNA levels of Nrf2 by binding to its promoter [28,29]. While some transcription factors (such as cFos, p53, p65, Fra1, Bach1, C/EB, ATF1, ATF3, estrogen receptors (ER), short-form estrogen-related receptor (SFERR), peroxisome proliferator activated receptor α (PPAR-**α**) and retinoic acid receptor, (RAR-α)) regulate Nrf2 transcription negatively, other transcriptional factors (such as JDP2, Jun, CBP, BRG1 and p21) can induce Nrf2 activation [21,30,31,32,33,34,35,36,37,38,39]. In addition, a nuclear receptor retinoic X receptor alpha (RXRα) was recently identified that interacts with the Neh7 domain of Nrf2 and, specifically, inhibits Nrf2 activity in the nucleus [40]. 

## 4. Nrf2 Function in Oxidative Stress and Toxicity

ROS and reactive nitrogen species (RNS) are continuously produced in humans from internal metabolism and external exposure [41]. Oxidant species are produced due to physiological action in order to control some vital procedures, such as cell division, inflammation, immune function, autophagy, and stress response [42]. Excessive production of oxidants can cause OS-related cellular damage and this process plays a critical role in the development of metabolic and chronic disease, such as cancer, autoimmune disorders, and neurodegenerative diseases [43,44]. 

Nrf2 acts as a mediator to induce drug-metabolizing enzymes (DMEs) with the help of species like antioxidants and electrophiles [45]. Research over the past decade demonstrates an important initial role for Nrf2 in OS resistance. Nrf2 is physiologically very effective against anticancer agents to protect cells from OS [46].

Nrf2, a transcription factor with a great affection to OS, binds to AREs in the nucleus and stimulates the transcription of many antioxidant genes. OS makes Nrf2 separate from Keap1 and to transfer into the nucleus, which results in its binding to AREs [47,48]. Excess Nrf2 has been confirmed to be cytoprotective in numerous tissues [49,50]. Heme oxygenase-1 (HO-1) is known as an Nrf2-dependent gene that mimics many critical properties of Nrf2 [51], which is responsible for eliminating toxic heme and produces biliverdin, iron ions and carbon monoxide. HO-1 and related molecules show beneficial effects via defense against oxidative damage and involvement to angiogenesis. Instabilities in the proper HO-1 level are linked to some disorders, such as neurodegeneration, cancer or macular degeneration. HO-1 contribution in cancer development is well recognized, but also HO-1 might be defensive for cancer cells in some tumor types [52]. HO-1 introduction stops cell transformation through an antioxidant defensive mechanism in healthy cells. Unfortunately, malignant cells benefit from HO-1 upregulation supporting tumor growth, invasion, and metastasis [53].

ROS and other endogenous reactive molecules may promote the release of Nrf2 that binds to the ARE in the nucleus. This binding motivates gene transcription and stimulates the antioxidant defenses [54,55]. The activation of Nrf2 has been thought to be responsible for the antioxidant action of many antioxidants capable of disrupting straight or ultimately the Keap1-Nrf2 complex [56].

Nrf2 is a main controller of the antioxidant reaction and xenobiotic metabolism via several of antioxidant and Phase II detoxification genes [57]. Nrf2 defends cells from ROS radiation, and toxic substances. Motivation of the Nrf2 pathway could be a promising approach for chemoprevention, as well as prevention against chronic diseases, such as cardiovascular disease, neurodegenerative diseases and pulmonary injury [58].

## 5. Nrf2 in Cancer

### 5.1. Molecular Basis of Nrf2 Activation in Cancer Cells

It is widely accepted that Nrf2 is an important player in the cellular defense mechanism that protects cells from cancer progression and promotes cell survival under stress conditions in normal cells (Figure 1A). However, emerging data indicate that the Nrf2 defense mechanism also shields tumor cells from chemotherapeutic agents, radiation therapy and anti-cancer drugs and aberrant elevation of Nrf2 causes therapeutic resistance and metastatic invasion of cancer cells [31] (Figure 1B). Increased Nrf2 levels have been shown in many clinical cancer studies including melanoma, lung, ovarian and endometrial carcinomas, pancreatic cancer, renal cancer, breast, colorectal cancer and hepatocellular carcinoma, and etc. Table 1 [59,60,61,62,63,64,65,66,67,68,69,70,71,72,73,74,75,76,77,78]. High level of Nrf2 activity in cancer cells reduces susceptibility to chemotherapeutic agents, and repression of Nrf2 reverses drug resistance and sensitivity against radiation therapy [75,79,80]. Nrf2-signaling pathway is compromised by multiple mechanisms including genetic, epigenetic and transcriptional changes.

#### 5.1.1. Somatic Mutations in Nrf2-Signaling Pathway

Somatic mutations in NRF2 (gain of function mutations) and KEAP1 (loss of function mutations) result in constitutive activation of Nrf2 and its target genes, and these mutations have been identified in many different cancers [62,69,75,81,82]. KEAP1 mutations occur more frequently than NRF2 and these mutations are found in different positions in the coding region. On the other hand, NRF2 mutations are located in KEAP1 binding motifs (DLG1 or ETGE motifs) of Neh2 domain [83]. KEAP1 mutations cause conformational changes that reduce its affinity for Nrf2 and lead to aberrant activation of Nrf2 [82]. Loss of exon 2 of NFE2L2 (Keap1 binding domain loss) was recently reported to be a new mechanism that also causes aberrant Nrf2 accumulation in the nucleus and promotes cell survival in lung, head and neck cancers [84]. Keap1 or Nrf2 independent mutations in oncogenes, including EGFR, Kras, Braf, Myc, and the Bcr-Abl fusion may also increase Nrf2 levels, resulting in ROS detoxification and chemo-resistance in cancer [29,85].

#### 5.1.2. Epigenetic Modifications in Nrf2-Signaling Pathway

Besides somatic mutations of KEAP1, epigenetic alterations in promoter regions of the KEAP1 gene can lead to aberrant activation and nuclear accumulation of Nrf2 protein in cancer cells. Hypermethylation of the promoter region of KEAP1 was reported in several cancers, including lung and prostate cancer, causing down-regulation of Keap1 expression and accumulation of Nrf2 [86,87]. On the other hand, demethylation of NRF2 promoter regions was shown in colon cancer cells, resulting in the upregulation of Nrf2 [88]. Because deregulation of the Nrf2 signaling is linked to chemo-resistance in a variety of tumor types, the reversal of KEAP1 methylation or NRF2 demethylation could be a novel strategy to increase anticancer drug sensitivity.

Micro RNAs (miRNAs) are small non-coding RNA molecules containing about 18–25 nucleotides that regulate the post-transcriptional activity of many genes by sequence-specific binding to mRNA [89]. Interestingly, recent studies have shown that microRNAs play a crucial role in the regulation of Nrf2 signaling. miR-144 was the first miRNA to be characterized as a negative regulator of Nrf2 expression in sickle cell disease [90]. miR-144 represses Nrf2 expression, together with its targets, such as superoxide dismutase 1, catalase, and glutamate-cysteine ligase subunits [21]. Similarly, overexpression of miR-28 in MCF7 breast-cancer cell lines decreased mRNA and protein levels of Nrf2 [91]. On the other hand, Nrf2 is upregulated indirectly by miR-200a that targets KEAP1 mRNA and leads to degradation [92]. These miRNAs can be potentially novel anticancer drug targets for preventing the deregulation of Nrf2-signaling pathway in cancer.

#### 5.1.3. Cooperation between Nrf2 and Other Proteins

Studies have demonstrated that different proteins in cancer progression can deregulate Nrf2 signaling by altering the Nrf2–Keap1 binding [93]. Nrf2 signaling is negatively regulated by p53, which suppresses Nrf2 target genes (such as x-ct, NQO1, and GST1) [94]. These results suggest that, under stressful conditions, induction of p53 inhibits Nrf2 by reducing antioxidant defense to promote cell death. On the other hand, p21, a direct downstream target of p53, regulates Nrf2 expression positively by disrupting Nrf2 and Keap1 binding [44] As a result, cytoprotective genes targeted by Nrf2 are reduced in the absence of p21, but these target genes are increased in the presence of p21 [39]. p62 is an adapter protein that binds to ubiquitinated substrates for selective autophagy [95]. Phosphorylated p62 binds to Keap1 and prevents to Nrf2 ubiquitination, causing Nrf2 stabilization. Importantly, abnormal accumulation of p62 is observed in studies about hepatocellular carcinoma [96], resulting in Nrf2 activity that could contribute to cancer progression.

It has been reported that Nrf2 level is also modulated through protein-protein interactions with Nrf2 or Keap1. Wilms tumor gene on the X chromosome (WTX) prevents Nrf2 degradation by binding to Keap1 [97]. Similarly, the partner and localizer of BRCA2 (PALB2) was also shown to promote nuclear accumulation of Nrf2 by suppressing Keap-1 mediated ubiquitination of Nrf2 [98]. Recently, the dipeptidyl peptidase III (DPP3) was identified as a Keap1 interacting protein, which blocks Nrf2-Keap1 interactions and activates Nrf2-dependent transcription in cancer cells [99]. Furthermore, the tumor-suppressor gene BRCA1 has been shown to activate Nrf2 signaling by physically binding to Nrf2 [39]. In another study, KAP1 (KRAB (Krüppel-associated box)-associated protein 1) was identified as a novel Nrf2-NT-interacting protein, which participates in the oxidative stress response by enhancing Nrf2-dependent transcription [100]. On the other hand, c-Myc was shown as an interaction partner of Nrf2, which reduced the half-life of Nrf2 [101]. Therefore, these interaction partners can be targeted in combination with Nrf2 for cancer treatment.

## 6. Nrf2 Inhibitors as Potential Antineoplastic Drugs

Some Nrf2 inhibitors have been stated for the treatment of Nrf2-addicted cancers. One of them is brusatol, which is a natural quassinoid. It was found that brusatol stimulates poly-ubiquitination of Nrf2, which decreases the Nrf2 protein level. The inhibitory effect of brusatol to Nrf2 is revealed to not be dependent of its repressor Keap1. Brusatol was found beneficial for the inhibition of Nrf2 signaling [102].

It has been established that stimulation of Nrf2 helps to lower chemo-resistance in NSCLC cells [103]. Many flavonoids are stated to induce Nrf2-dependent gene expression in several cancer cell lines [104]. Luteolin was found as an inhibitor of antioxidant response element-driven gene expression. Using non-small-cell lung cancer cell lines (A549 cells), which have active Nrf2, luteolin provoked an intense decrease in Nrf2 at both the mRNA and the protein levels. Moreover, luteolin considerably sensitized A549 cells to the neoplastic drugs oxaliplatin, bleomycin, and doxorubicin [105].

Another Nrf2 inhibitor, halofuginone, was established to develop a chemosensitizing influence on Nrf2-addicted cancer cells [106]. In this study, halofuginone was shown to decrease Nrf2 at protein level, through suppression prolyl-tRNA synthetase activity. A chalcone derivative 4-methoxy-chalcone (4-MC) showed suppression on the transcriptional activity of Nrf2 in A549 cells but to activate it in HEK293 cells. 4-MC was also found to down-regulate expression of Nrf2. Inhibition of Nrf2 signaling has effects on the enhanced production of ROS. Use of 4-MC was found promising to improve the sensitivity of tumor cells to the pharmacological effect of cisplatin over the regulation of Nrf2/ARE signaling [107]. Bardoxolone methyl (BM) resembles the natural product oleanolic acid is a semisynthetic triterpenoid effectively induces Nrf2 and has been examined in different types of cancer, such as leukemia and some solid tumors [108,109]. BM display Michael acceptor activity and act as the most potent inducers of Nrf2 [110]. Camptothecin is another novel Nrf2 inhibitor that might be suggested in combination with other anticancer drugs to improve their effectiveness in treating high Nrf2-expressing cancers. It was found that camptothecin evidently inhibited Nrf2 expression and transcriptional action in different types of cancer cell lines, including HepG2, SMMC-7721 and A549 [111].

The effective protection activity of Nrf2 has been stated generally during tumor initiation. However, it is now well recognized that Nrf2 shows a dual effect in carcinogenesis. Growing number of research showed the oncogenic properties of Nrf2 in lung cancer, esophagus and skin and renal cell cancer [40,73,112,113,114].

Nrf2 is found in practically all cell types and tissues, but it is prominent in tissues where main detoxification reactions take place, such as intestine, lung, and kidney. It has a protective role against oxidative damage and carcinogenesis through binding to AREs [115].

Recent studies specify that targeting Nrf2 may be a new therapy to diminish tumor and develop a defense. It has been established that Nrf2 is defending normal cells under OS. The excess Nrf2 in tumor cells carry on its defense toward cytoprotection. This might help to defend tumor cells against OS. This process by which cancer cells adjust themselves to survive in augmented OS and resist treatment is called “redox adaptation”. Therefore, it is debatable whether the activation, or the inhibition, of Nrf2 is beneficial for the prevention or treatment of cancer [116].

Numerous compounds modulate the Nrf2 pathway to perform anticancer activity. It is obvious that both Nrf2 inducers and inhibitors could be valuable as anticancer policy. Nevertheless, due to modulating effects of Nrf2, it is active in the detoxification procedure of anticancer drugs, and its activation in cancer cells possibly will lead to chemo-resistance. A beneficial or unfavorable process of Nrf2 in cancer cells basically depends on the close control of its action, surroundings of tumor and cell type [117]. Over the last decade, Nrf2 has been known as a critical biomarker in cancer prognosis and therapy [118]. High expression of Nrf2 in cancer patients did not get the highest benefit from anticancer drugs and radiotherapy [119]. A risk-score system established on Nrf2-mediated gene expression was established to provide estimation on recurrence-free survival and overall survival in cancer patients [120]. It is crucial to find out the genetic polymorphisms in the NRF2 gene in cancer patients before their cancer treatment in the clinic [121]. In some tumors, Nrf2 is determinedly stimulated because of somatic mutations in either Nrf2 or Keap1, and, therefore, upholding tumor development and resistance to oxidants and anticancer drugs [29,122]. Nrf2 mutations that allow this transcription factor to avoid Keap-1-mediated repression have been found in 10% of patients with lung cancer [123]. In some cases, higher quantities and activity of Nrf2 reduces the effectiveness of some anticancer drugs, such as carboplatin, cisplatin, 5-fluorouracil, and doxorubicin [124].

Nrf2 is crucial to providing defense against OS and also preventing tumor promotion and progression [125,126]. Studies showed that Nrf2-knockout organisms display bigger injury in vital organs after exposition to toxic substances. Lack of Nrf2 develops weakness to experimental ischemic acute kidney injury. Increasing Nrf2 motivation by synthetic triterpenoid CDDO-imidazolide showed that treated mice had enhanced renal histology with a reduction in tubular damage, as well as a lessening in pro-inflammatory cytokine and chemokine construction [126].

The role of Nrf2 in defense against toxic effects of chemicals was proven on Nrf2 knockout mice using acetaminophen (*N*-acetyl-4-aminophenol (APAP)) [127]. The mice were orally given a single dose of APAP at 0, 150, 300, or 600 mg/kg. Doses of 300 mg/kg APAP or higher doses caused death in the homozygous knockout mice only. The surviving mice exhibited more brutality in hepatic injury than the wild-type mice, as confirmed by improved plasma alanine aminotransferase activity, reduced hepatic non-protein sulfhydryl content, and centrilobular hepatocellular necrosis. The data demonstrated that Nrf2 has a defending role against APAP hepatotoxicity.

Nrf2 stabilizes oxidative tissue damage and inflammation through transcriptional activation via the ARE. Protecting activity of Nrf2 in the development of emphysema was observed [128] by testing cigarette smoke-induced Nrf2-knockout mice. Emphysema was first detected at 8 weeks and worsened by 16 weeks following cigarette smoke-exposure, while no pathological anomalies were detected in the control group. The results show that Nrf2 is able to defend against the development of emphysema by adjusting the oxidant/antioxidant balance.

Citrus coumarin auraptene was studied in order to identify the possible effect on premalignant mammary lesions via activation of Nrf2/ARE [129]. While the mice introduced with carcinogen displayed premalignant wounds, Nrf2 knockout mice showed significant proliferation in mammary carcinoma growth percentage. There was no significant difference in general survival, but the Nrf2 knockout mice had significantly lower mammary tumor-free survival. It is noteworthy that the frequencies of lung adenomas in the Nrf2 knockout mice were very much higher than in the control group.

ROS and RNS are believed to be a main cause underlying the contribution of chronic inflammation to cancerogenic alteration [130]. Nrf2 shows an important role in defending many tissues against inflammation, which is a probable treatment for colorectal and many other cancers. Nrf2 supports defense against dextran sulfate sodium (DSS)-induced colitis/inflammation and protects against inflammation-associated colorectal carcinogenesis [131]. This result was supported by an experiment using azoxymethane/DSS-treated Nrf2 knockout mice [132]. Nrf2 knockout mice augmented occurrence, and size of all colorectal tumors, including adenomas, versus treated wild-type mice. The knockout mice also had augmented cyclooxygenase-2 and 5-lipoxygenase expressions, and prostaglandin E2 and leukotriene B4 levels in tumor. These results represented that Nrf2 has a significant importance in defending against colorectal cancer.

The pharmacological significance of Nrf2 has been revealed by many studies mainly using Nrf2-deficient mice and research of single nucleotide polymorphism in the NRF2 gene [133]. Nrf2 activation successfully avoids chemical carcinogenesis by modulating antioxidant and detoxification abilities and makes anticancer immunity. Nrf2 successfully inhibits the action of myeloid-derived suppressor cells and stops apoptotic Treg cell-mediated immunosuppression by defending Treg cells from apoptosis [134,135]. Various clinical studies have revealed strong correlations between Nrf2 stimulation in tumor tissues. Data show Nrf2 increase generally related with poor diagnosis in many cancer types. Somatic mutations of NRF2 are also prognostic markers of many cancer types like non-small cell lung cancers, esophageal cancers, and head and neck cancers [71,136].

Since Nrf2 shows boundless benefits on cancer cells, including therapeutic resistance, improved antioxidant capability and aggressive tumorigenic ability, cancer cells with Nrf2 activation often develop “Nrf2 addiction”. Even though constant stimulation of Nrf2 helps growth and survival benefits on cancer cells, high stimulation of Nrf2 in normal cells is quite toxic. These findings suggest that definite requirements allow for the formation of Nrf2-addicted cancers [137].

Some Nrf2 inhibitors have been stated for the treatment of Nrf2-addicted cancers. Brusatol, a natural quassinoid, stimulates poly-ubiquitination of Nrf2 and decreases the Nrf2 protein level [102]. Halofuginone creates a chemosensitizing action on Nrf2-addicted cancer cells. Nrf2 protein level is decreased by halofuginone, which is consistent with a short half-life of the Nrf2 protein [106]. Development of anticancer immunity in cancer-bearing hosts has been revealed to be very active for eliminating cancers since Nrf2 stimulation inhibits immunosuppressive procedures made by myeloid-derived suppressor cell and apoptotic Treg cells [135]. Cancer therapy with Nrf2 inducers may cause potential malignant development consequences of Nrf2 stimulation in cancer cells. Thankfully, the effects of Nrf2 inducers on Nrf2-addicted cancer cells are estimated to be insignificant.

Inhibition of Nrf2 is an encouraging approach for the treatment of Nrf2-addicted cancers. Nevertheless, using systemic Nrf2 inhibitors may have unwanted effects on cancer-bearing hosts, seeing the essential roles of Nrf2 in cytoprotection. Finding novel therapeutic targets besides Nrf2 for Nrf2-addicted cancers have been still under investigation.

## 7. Challenges in Nrf2 Inhibitor Drug Development

It is known that one of the chief pathways in charge for cell protection against OS is the Nrf2/Keap1-signaling pathway [81]. Nrf2 has been commonly believed as a cytoprotective transcription factor, and additionally, a tumor suppressor. Certainly, at lower levels, Nrf2 is able to remove ROS, carcinogens [138]. However, there are various publications signifying that the stimulation of Nrf2/Keap1-signaling pathway is not beneficial in all cancer types and stages [138,139]. Some studies showed that, the augmented Nrf2 activity in several cancer types supports malignant cells in defending against OS and anticancer drugs. In some cases, Nrf2 supports in avoiding apoptosis via stimulation of cytoprotective genes [138]. Oncogenic character of Nrf2 at the early stages of cancer encourages the researchers to design new inhibitors of Nrf2/Keap1-signaling pathway. Though, there are big numbers of “reactive” indirect Nrf2 modulators, their pharmacological activity is not satisfactory and very limited due to “off-target” side effects caused by the attack on cysteine residues of other important cellular proteins [140].

The characterization of the crystal structure of Keap1 in complex with the Neh2 domain of Nrf2 may deliver chances to design molecules that specially and selectively interfere with the binding of Keap1 and Nrf2 [141]. Nrf2 has a very short half-life, even after drug-induced stabilization. For this reason, the most appropriate dosing schedule for a medicinal benefit needs to be conditional by indirect indicators of its activation in the diseased organs [142]. There are some questions related to the NRF2 inhibitors continue and must be resolved before more drugs are applied in anticancer therapy. Nevertheless, challenges regarding target specificity, pharmacodynamic properties, efficacy and safety remain. 

## 8. Conclusions

Nrf2 has a significant part in cellular defense to OS and exogenous toxic materials, and it is strictly associated to inflammatory reactions, respiratory system diseases, cardiovascular diseases, and malignant tumors [47]. ROS elimination by the Nrf2-mediated induction of target genes could be one way to explain the activity of chemo-preventive compounds, which are able to prevent or delay the existence of malignancy [50].

OS is associated with cancer initiation and progression. The Nrf2 transcription factor is the main regulator of antioxidant genes and has a critical role in regulating the metabolic pathways important in cancer cells. Undeniably dissimilar results show that there are beneficial and harmful effects of targeting Nrf2 in some cancer cells. Antioxidant agents may help explain the activity of Nrf2 in cancer, as well as its power as a biomarker of cancer progression and therapy.

Both Nrf2 inducers and inhibitors could be beneficial in cancer treatment approaches. Nonetheless, Nrf2 is able to modulate many systems possibly involved in the detoxification procedure of anticancer drugs; its activation in cancer cells may cause chemo-resistance. The beneficial or disadvantageous role of Nrf2 in cancer cells basically depends on the constricted control of its action [118]. Discovery, development and improvement of Nrf2-centered approaches are critical and challenging tasks for the treatments of cancer.

## Figures and Tables

**Table 1:** The collection of clinical studies related to the role of Nrf2 in various cancer types.

Type of Cancer	Number of Patients	Conclusion	Ref.
Brain Glioma	75	The expression of Nrf2 and p62 was associated with tumor grade and survival in patients with gliomas	[64]
Bladder cancer	44	Nrf2 expression is associated with short overall survival in bladder cancer patients	[65]
Breast cancer	106	Nrf2 protein plays important roles in the proliferation and/or progression of breast carcinoma	[66]
Cervical cancer	89	Strong nuclear expression of NRF2 was significantly associated with reduced cytoplasmic Keap1 expression in cervical cancers due to hypermethylation.	[67]
Colorectal cancer	76	Nrf2 was highly expressed in CRC tissues compared with adjacent non-tumor tissues	[68]
Esophageal squamous cell carcinoma	82	Oncogenic Nrf2 mutation induces dependence on the mTOR pathway during carcinogenesis	[69]
Gastric cancer	175	Nrf2 expression is closely associated with clinicopathological factors and the prognosis of gastric cancer patients	[70]
Head and neck squamous cell carcinoma	302	Nrf2 activation is potentially clinically relevant as a prognostic indicator in HNSCC	[71]
Hepatocellular carcinoma	65	Nrf2 was up-regulated in HCC, and expression of Nrf2 was correlated with tumor differentiation metastasis, and tumor size	[72]
Non-small cell lung cancer	443	NRF2 regulates serine biosynthesis in non-small cell lung cancer	[73]
Melanoma	121	Nrf2 influences prognosis in melanoma	[74]
Ovarian cancer	64	Nrf2 may serve as an important therapeutic target for novel drugs capable of preventing or reversing resistance to chemotherapy in ovarian cancer	[75]
Pancreatic adenocarcinoma	103	Nuclear Nrf2 expression is related to a poor survival in pancreatic adenocarcinoma	[76]
Renal cell cancer	89	Keap1/Nrf2 axis deregulation is an important prognostic marker in renal cell carcinoma	[77]
Tyroid carcinoma	42	Nrf2 pathway has potential diagnostic, prognostic, and/or therapeutic utility in papillary thyroid carcinoma	[78]

## Abbreviations

APAP	*N*-acetyl-4-aminophenol
ARE	Antioxidant response element
CREB	cAMP Response element binding protein
CHD6	Chromo-ATPase/helicase DNA binding protein 6
CNC-bZip	Cap’n’collar type of basic region leucine zipper factor family
DME	Drug metabolizing enzymes
ER	Estrogen receptors
GCL	Glutamate-cysteine ligase
GSK-3β	Glycogen synthase kinase 3
GSTs	Glutathione S-transferases
HMOX1	Heme oxygenase-1
HO-1	Heme oxygenase
Keap1	Kelch like ECH associated protein
NQO-1	NADPH quinone oxidoreductase
Nrf2	Nuclear factor-erythroid 2 p45-related factor 2
OS	Oxidative stress
PPAR-α	Peroxisome proliferator activated receptor α
RAC	Receptor-associated co-activator
RAR-α	Retinoic acid receptor
RXR	Retinoid X receptor alpha
RNS	Reactive nitrogen species
ROS	Reactive oxygen species
SFERR	Short-form estrogen-related receptor
β-TrCP	β-Transducin repeat-containing protein
Treg	Regulatory T cells
